# Use of Selective Serotonin Reuptake Inhibitor and Midodrine in a Patient With Autonomic Instability 2/2 Compressive Squamous Cell Carcinoma and Pain

**DOI:** 10.1177/2324709617749621

**Published:** 2018-01-29

**Authors:** Kyle Ball, Thomas P. Vacek

**Affiliations:** 1 Wright State University, Dayton, OH, USA

**Keywords:** SSRI, midodrine, neck malignancy, glossopharyngeal neuralgia, syncope

## Abstract

A rare cause of reflex syncope is metastatic cancers involving the head and neck. These can irritate the glossopharyngeal nerve and lead to glossopharyngeal neuralgia with associated syncope. This type of syncope is difficult to treat since it commonly involves both a vasodepressor and cardioinhibitory response, and typically requires removal of the irritative focus. We report a case of a 52-year-old male who presented from home with syncope. He endorsed a 5-week history of progressively worsened positional headaches and dramatic 40-pound weight loss with night sweats over 6 months. In the emergency department, his heart rate was noted to drop into the 20s with associated hypotension 60/31 mm Hg. Heart rate and blood pressure increased with intravenous atropine. Physical examination revealed a large ulcerative lesion in the left tonsillar area. After biopsy of the lesion, a diagnosis of stage IV squamous cell carcinoma of the neck was made; computed tomography angiogram and positron emission tomography/computed tomography confirmed involvement in the posterior tongue extending to the left palatine tonsil in addition to the left jugular chain. The patient was started on cisplatin and radiation therapy, but continued to have episodes of syncope associated with bradycardia and hypotension. After a failed trial of benztropine, the patient was started on sertraline and midodrine with resolution of syncope. This could be a potential treatment option in those with compressive mixed syncope who are not candidates for surgery or chemotherapy or are awaiting definitive treatment.

## Introduction

Syncope is a major cause of morbidity and mortality, with nearly 40% of the population having experienced at least one episode.^1^ It can be divided into several different broad categories, which include orthostatic, cardiogenic, neurogenic, neurocardiogenic, metabolic. The most common type is neurocardiogenic or reflex syncope. Reflex syncope is composed of vasovagal syncope, situational syncope, carotid sinus syndrome, and glossopharyngeal neuralgia (GPN) in some instances.^2^ Carotid sinus syndrome and GPN-associated syncope can be easily confused, making diagnosis and treatment a challenge. In this case, we present a patient with the onset of syncope related to newly detected neck cancer. To our knowledge, it is only the second case report in the literature describing onset of syncopal episodes associated with neck cancer with position of the neck. Moreover, this report serves to confirm the success of a lesser used therapy that currently has IIb recommendations for general use and may not otherwise be used in this context.

## Case Report

A 52-year-old male with a 5-week history of severe headaches was admitted after he had a syncopal episode while at home. He found himself down on the ground, and he was unaware of the initiating circumstances. There was no post-event confusion, weakness, tongue biting, incontinence of bowel or bladder, or any reported head trauma after the incident. This was the first such episode he was able to infer that occurred after having found himself on the floor. His headache was worse in the left temporal region and radiated through his neck, progressively increasing in severity over time. His headaches were described as severe, especially when he turned his head to the left. Over the past several months, he noted occasional drenching night sweats and a 40-pound unintentional weight loss over 6 months. Past medical history is significant for smoking 2 packs per day for the past 30 years, but otherwise he has no chronic diseases and takes no medications regularly.

While in the emergency department, he had several episodes of bradycardia noted on telemetry and had accompanying hypotension with values reaching as low as 60/31 mm Hg at one point. He was dizzy during these episodes, and both his blood pressure and heart rate responded to 0.5 mg of intravenous atropine. An electrocardiogram obtained during one of the episodes showed sinus bradycardia, ventricular rate of 25 beats per minute, with no signs of atrioventricular dysfunction or ischemia.

Physical examination revealed a large ulcerative lesion in the left tonsillar area with tenderness in the left submandibular region. No lymphadenopathy was appreciated in the neck. The rest of his examination was grossly normal. His heart rate and blood pressure, measured after an episode had resolved, was 93 beats per minute and 113/61 mm Hg, respectively; vital signs were otherwise normal. Routine laboratory investigation was normal. A computed tomographic angiography of the head and neck showed extensive soft-tissue swelling of the left palatine tonsil and surrounding tissues. Otolaryngology was consulted, and a biopsy of the left tonsillar mass was obtained. The histology of the left tonsillar lesion was consistent with invasive squamous cell carcinoma and was P16 positive on immunostain. A subsequent positron emission tomography/computed tomography showed hypermetabolic regions in the posterior tongue extending to the left palatine tonsil, in addition to metastatic involvement of left jugular chain. He was diagnosed with stage IVa squamous cell cancer with nodal involvement of the left jugular chain. Echocardiography failed to show any abnormalities. Interestingly, most of his episodes of bradycardia and hypotension were preceded by changes in neck position (he had repeated episodes of bradycardia when he moved his neck in certain ways), which was initially attributed to a vasovagal response related to pain.

The patient continued to have repeated episodes of symptomatic hypotension and bradycardia throughout his stay. He was started on a trial of benztropine (2 mg every 8 hours initially) to help ameliorate these symptoms. After increasing the dose over several days, he continued to have episodes of hypotension and bradycardia and was ultimately started on a dopamine drip. He continued to require dopamine infusions intermittently, and phenylephrine was added on as well. Intravenous atropine continued to be the only treatment to consistently provide relief. After failing to improve on the benztropine, he was started on midodrine (5 mg twice daily) and sertraline (50 mg daily).

Once being started on this regimen, he had no further episodes of bradycardia and required no more atropine. Although he had several episodes of orthostatic hypotension, complicated by acute blood loss anemia, he was able to eventually come off the dopamine and phenylephrine drip completely once the bleeding was stopped. Also, he began treatment with high-dose cisplatin 1 week into his stay, and started a course of 35 rounds of radiation therapy 2 weeks into his stay. He remains on midodrine, sertraline, and cisplatin every 3 weeks, and he is over halfway through radiation therapy without any recurrences of syncope.

This patient’s picture was consistent with a small number of case reports describing neurocardiogenic syncope secondary to head and neck cancer. In this case, it was thought to be due to irritation of the glossopharyngeal nerve with spread of the cancer to the jugular chain.

## Discussion

GPN is a rare cause of head and neck pain. Although the exact mechanism of the syncope is not defined, it is thought to be similar to that of carotid sinus hypersensitivity (CSH). This can make it difficult to distinguish these 2 etiologies, but one major factor for diagnosis of CSH is induction of asystole or hypotension on carotid sinus massage,^3^ which was not present in our patient. The discussion will focus on the etiology, pathophysiology, and treatment of syncope associated with GPN, particularly in conjunction with tumors of the head and neck.

Typically, the condition occurs after age 40 due to abnormally tortuous arteries that compress the nerve. GPN can be divided into primary and secondary etiologies. These secondary causes include vascular tortuosity (as previously mentioned), intracranial tumors near the cerebellopontine angle or cranial base, parapharyngeal abscess, nasopharyngeal and laryngeal tumors, trauma, Eagle’s syndrome (elongated styloid process), occipito-cervical cranial abnormalities, or inflammatory processes.^2,4^ It has been described as a cause of syncope in 1% to 2% of those affected.^5^

In order to understand the pathophysiology of reflex syncope, one must have a firm grasp on the anatomic relations in the head and neck. There are 2 main baroreceptors: the carotid body and the aortic body. These are stretch receptors located in the carotid arch and aortic sinuses, respectively. Increased stretch in the baroreceptors can be caused by either extrinsic or intrinsic pressure. After stimulation, afferent impulses are carried along the glossopharyngeal and vagus nerve to the vasomotor center of the medulla oblongata. The glossopharyngeal nerve travels into the skull through the jugular foramen, after coursing along the jugular chain in the neck.^2,6^ It was thought that tumor invasion to the left jugular chain was the irritative focus in our patient’s case. There are 2 efferent pathways involved in the reflex arch after stimulation of the stretch receptors occurs. The first is stimulation of parasympathetic impulses carried down the vagus nerve with resultant decreased heart rate (cardiodepressor effect). The second path involves inhibition of efferent sympathetic impulses leading to decreased vascular tone.^5^ Syncope occurs when these counterbalancing effects are hyperactive or the body cannot properly compensate for changes in hemodynamic status. Treatments are generally aimed at combating these 2 deleterious mechanisms.

Since a tumor can affect this reflex arc proximally (such as the glossopharyngeal nerve), it is thought that it predominantly has both a cardioinhibitory component and a vasodepressor component.^7^ There have been several mechanisms proposed for tumors or masses causing syncope. One theory is that direct pressure exerted on the glossopharyngeal nerve can cause hypersensitivity. Another idea involves permanent depolarization of axons, which leads to a lower threshold for depolarization. Last, the compression could cause an irritative focus that leads to repeated stimulation.^8^ This may be why in this case we saw hemodynamic changes that involved motion of the neck and sometimes were independent of that.

Treatment for GPN described in literature focuses mostly on treatment of pain associated with neuralgia and removal of the compressive force on the nerve. Since there is similarity between trigeminal neuralgia and GPN, the treatment parallels that of each other. Given the rarity of GPN, and the infrequency of which it is associated with syncope, there are not many studies available that evaluate treatment options.

The first-line agent remains anticonvulsants, particularly carbamazepine for symptoms of neuralgia.^4^ Other commonly used medications include gabapentin, pregabalin, phenytoin, or oxcarbazepine. In those with the additional cardiac symptoms, atropine remains the first-line treatment during acute attacks (although this will not decrease the associated pain). For ongoing treatment, continued treatment with carbamazepine has been shown to stop episodes of syncope as well.^9^ In our particular patient, other options were utilized.

Benztropine was attempted initially with similar mechanism to droxidopa (increase dopamine availability) that is a current class IIa recommendation by the American College of Cardiology for those with neurogenic component of syncope. The patient eventually received maximum dose after titration to see if there was any benefit. Because of the intermittent nature of the episodes during the hospital course, the psychological principle of variable reinforcement may have been the reason we continued our course of titrating this drug. Eventually, we realized that it did not reduce the number of episodes over the course, so it was abandoned. Midodrine is a class IIa drug to give that could address vasodepressor response, but would not have had benefit for the cardioinhibitory response. Hence, we wanted to try another drug that could have at least some chance in reducing cardioinhibitory response at the same time that a more highly recommended drug to treat vasodepressor response was given, midodrine.

Selective serotonin reuptake inhibitors (SSRIs) can be an effective treatment for reflex syncope that is refractory to other lines of therapy. Paroxetine (20 mg) has been studied in vasovagal syncope and in one randomized, double-blind, placebo-controlled study showed spontaneous syncope rates of 17.6% versus 52.9% in the treatment and control group, respectively.^10^ Other SSRIs shown to have a benefit in this group include sertraline 50 mg and fluoxetine 20 mg daily.^10^ This could potentially be of benefit in those with GPN-related syncope given the similarity in pathophysiology, but not much data are available at this time.

Midodrine hydrochloride, an α-1 agonist, has also been used for syncope, particularly in reflex-mediated syncope and symptomatic orthostatic syncope. One study, evaluating 11 randomized controlled studies in treatment of these conditions, found that midodrine could have a positive impact of clinically relevant outcomes without causing any serious adverse events. One drawback, though, was the significant amount of side effects related to the drug’s mechanism, such as urinary retention, pilomotor erection, and systolic hypertension.^11^

In this case, the patient stopped experiencing episodes of bradycardia after being started on sertraline and midodrine. His persistent orthostatic hypotension (complicated by acute blood loss anemia) required additional agents such as fludrocortisone, sudafed, and compression stockings. After this issue was addressed, he eventually was able to come off pressors completely. This argues that there could be a potential benefit derived from the sertraline and midodrine combination in this patient population, which has yet to be thoroughly studied.

Pacemakers have been the cornerstone in treatment of CSH. This is appropriate since CSH is predominantly a cardioinhibitory response. However, when used alone, their success in preventing syncope in GPN is much lower. In one study, by Tulchinsky and Krasnow,^12^ pacemakers were evaluated in patients with syncope and associated malignancy of the head and neck. Pacemakers alone alleviated syncopal symptoms in only 1/11 patients; however, when combined with an α-agonist, this increased to 4/5 patients.^12^ Although these data are small, it further supports that GPN-related syncope in head and neck cancers involves both a vasodepressive response and a cardioinhibitory response.^12^ Pacemaker implantation was deferred in this particular case due to the vasodepressive component of our patient’s syncope. The question remains whether this could be a viable option if combined with α-agonists.

Surgical treatment options are typically reserved for those who fail conventional therapies when concerning GPN. One method involves sectioning out of the nerve of Hering or the glossopharyngeal nerve.^13^ These are applied to patients with GPN only; other than complete resection of the tumor, it may not be of substantial benefit in patients presenting with head and neck cancer. Several studies have looked at success of surgical interventions in patients with cancer as the cause of syncopal episodes. Surgery in our particular case would have been a difficult endeavor, given the delicate nature of the surrounding tissues in the neck and the extent of spread.

In one study by Dykman et al,^14^ abolishment of the greater palatine nerve had cessation of symptoms in those with malignancy associated with this nerve. Another study showed that vascular decompression of the greater palatine nerve fixed symptoms of both pain and syncope.^15^ Similar to surgery, chemotherapy and radiation could work in a comparable fashion, by relieving the compressive force. Most cases involving head and neck cancer would benefit from a multifaceted approach involving both medications and chemoradiation.

## Conclusion

It is difficult to tell if the pain felt by this patient was true GPN or due to direct invasion of the tumor. Regardless, it is relatively certain that his syncope was a direct result of malignant irritation of the glossopharyngeal nerve leading to reflex-mediated syncope. The definitive treatment for patients with head and neck cancer would be removal of the irritative focus, whether through chemotherapy and radiation or surgical intervention.

In our patient, his improved symptoms could be attributed to the midodrine-sertraline combination, although there are little data to support the pharmacologic combination in head and neck cancer. For those patients who may not be immediate candidates for chemotherapy, radiation, or surgery, these drugs could be particularly beneficial. Most studies have shown roughly 1- to 2-week period before chemoradiation effects are noted, and the SSRI-α-agonist combination could be of morbidity benefit in the interim, with the advantage of relatively safe side effects. Another potential option that could provide benefit includes anticonvulsants, such as carbamazepine, although this was not attempted in our patient.

We may need further research to determine treatment options in those with head and neck cancer who are not candidates for surgical or chemoradiation therapy. Currently, there is abundant research on CSH and GPN, but lacking are longitudinal studies in those with head and neck cancer. Potential options include midodrine, anticonvulsants, α-agonists, and SSRIs alone or in combination with pacemakers. This could be of palliative benefit to this patient population, especially in those who are not amenable to radiation therapy, chemotherapy, or surgical interventions.
